# Involuntary sustained firing of plantar flexor motor neurones: effect of electrical stimulation parameters during tendon vibration

**DOI:** 10.1007/s00421-020-04563-7

**Published:** 2021-01-03

**Authors:** Ricardo N. O. Mesquita, Janet L. Taylor, Benjamin Kirk, Anthony J. Blazevich

**Affiliations:** 1 Centre for Exercise and Sports Science Research, School of Medical and Health Sciences, Edith Cowan University, Perth, Australia; 2 Neuroscience Research Australia, Sydney, Australia

**Keywords:** Motor neurone, Central torque, Bistability, Serotonin, Noradrenaline

## Abstract

**Purpose:**

Simultaneous application of tendon vibration and neuromuscular electrical stimulation (NMES) induces an involuntary sustained torque. We examined the effect of different NMES parameters (intensity, pattern of stimulation and pulse width) on the magnitude of the evoked involuntary torque.

**Methods:**

Plantar flexor torque was recorded during 33-s Achilles tendon vibration with simultaneous 20-Hz NMES bouts on triceps surae (*n* = 20; 13 women). Intensity was set to elicit 10, 20 or 30% of maximal voluntary contraction torque (MVC), pulse width was narrow (0.2 ms) or wide (1 ms), and the stimulus pattern varied (5 × 2-s or 10 × 1-s). Up to 12 different trials were performed in a randomized order, and then repeated in those who produced a sustained involuntary torque after the cessation of vibration.

**Results:**

Six of 7 men and 5 of 13 women produced a post-vibration sustained torque. Eight of 20 participants did not complete the 30% trials, as they were perceived as painful. Torque during vibration at the end of NMES and the increase in torque throughout the trial were significantly higher in 20 than 10% trials (*n* = 11; 9.7 ± 9.0 vs 7.1 ± 6.1% MVC and 4.3 ± 4.5 vs 3.6 ± 3.5% MVC, respectively). Post-vibration sustained torque was higher in wide pulse-width trials (5.4 ± 5.9 vs 4.1 ± 4.3% MVC). Measures of involuntary torque were not different between 20 and 30% trials (*n* = 8).

**Conclusion:**

Bouts of 5 × 2-s NMES with wide pulse width eliciting 20% MVC provides the most robust responses and could be used to maximise the production of involuntary torque in triceps surae.

## Introduction

Neuromuscular electrical stimulation (NMES) and tendon vibration have been used independently (e.g. Magalhães et al. 2013; Bochkezanian et al. 2018b) and simultaneously (Magalhães and Kohn 2010; Trajano et al. 2014; Bochkezanian et al. 2018a; Kirk et al. 2019) to induce motor unit (MU) activation, and hence muscle contractions. This involuntary MU activation is induced via both peripheral and central pathways. NMES depolarizes both motor and sensory axons (Bergquist et al. 2011b), inducing direct MU recruitment with signals travelling from the stimulation site to the muscle as well as an indirect MU recruitment with signals travelling through the spinal motor neurones (MNs). Tendon vibration imposes repetitive, small muscle-length changes that stimulate muscle spindles, inducing excitatory drive primarily from Ia-afferents onto the MN and provoking a reflexive activation of MUs (Grande and Cafarelli 2003).

In previous studies, an NMES-induced involuntary “extra torque” that is larger than would be expected from the direct activation of motor axons has been observed (e.g. Collins et al. 2001; Wegrzyk et al. 2015). The contribution of peripheral (i.e. within the muscle) mechanisms to this phenomenon is controversial, given that this “extra torque” was abolished during anaesthesia of the motor axons proximal to the stimulation site in some (Collins et al. 2001; Blouin et al. 2009; Lagerquist et al. 2009) but not all (Frigon et al. 2011) studies. Other empirical evidence corroborates the contribution of centrally-mediated mechanisms. This evidence includes greater levels of “extra torque” (Collins et al. 2002; Lagerquist and Collins 2010) and electromyographic (EMG) activity (Arpin et al. 2019) during wide- than narrow-pulse NMES, and the enhancement of H-reflexes, V- and F-wave amplitudes in association with extra torque development during NMES (Klakowicz et al. 2006; Lagerquist and Collins 2010; Bergquist et al. 2011a; Vitry et al. 2019) but also during tendon vibration (Magalhães et al. 2013) or both simultaneously (Magalhães and Kohn 2010). The relative contribution of peripheral and central pathways to this phenomenon has mainly been discussed in relation to NMES, and a greater central contribution is expected when combining both NMES and tendon vibration. Tendon vibration per se recruits low-threshold MUs (Grande and Cafarelli 2003) and the superposition of bursts of NMES may activate additional afferent fibres and consequently activate a greater proportion of the MU pool (Collins et al. 2001).

The central mechanisms commonly suggested to explain the occurrence of “extra torque” are a presynaptic development of post-tetanic potentiation of neurotransmitter release due to a repetitive depolarization of Ia afferents (Hirst et al. 1981) and/or postsynaptic activation of persistent inward currents (PICs). PICs are a strong intrinsic MN property activated via voltage-dependent channels on the MN dendrites (Hounsgaard and Kiehn 1993; Lee and Heckman 1996) and modulated by serotonergic and noradrenergic drive onto the MN (Lee and Heckman 2000). They amplify and prolong the effects of synaptic input (Heckman et al. 2005) by providing a sustained depolarising current to the MNs, accelerating initial MN firing and contributing to the repetitive firing required for muscle contractions (Heckman et al. 2008). As PICs are initiated by excitatory synaptic signals onto the MN, they can be initiated by excitatory afferent input (Heckman et al. 2008). Some features of responses to combined tendon vibration and NMES are consistent with the behavior of PICs. Self-sustained torque observed after the end of such trials (or after vibration alone; Gorassini et al. 2002) could be explained by PIC-related bistable behavior in some spinal MNs (Lee and Heckman 1998). The progressive increase of torque reported during vibration plus NMES (Magalhães and Kohn 2010; Trajano et al. 2014; Kirk et al. 2019) is similar to the warm-up effect that is thought to reflect depolarization-induced facilitation of voltage-gated calcium channels (Svirskis and Hounsgaard 1997). Finally, the “extra torque” is joint angle (i.e. muscle length) dependent (Trajano et al. 2014), consistent with the effect of reciprocal inhibition shown in vivo using voltage clamp techniques (Hyngstrom et al. 2007). Thus, it is possible that responses to combined tendon vibration and NMES are explicable by PIC activity, although a robust examination of these behaviors remains to be conducted to determine whether PIC strength can be estimated in vivo using this technique.

The magnitude of involuntary torque produced during simultaneous tendon vibration and NMES may hypothetically allow the indirect estimation of PIC behavior, presumably in a relatively large number of motor units in the absence of voluntary corticospinal drive and using equipment that is available in many laboratories. It might therefore prove to be a useful alternative to the paired motor unit technique (Gorassini et al. 2002), the standard method to indirectly estimate PIC strength in humans. Nonetheless, the technique under examination in this study has been rarely used in research and the specific test parameters that might provide the greatest involuntary torque have not been determined. These parameters should be optimized before further investigations are conducted. Whilst the tendon vibration parameters that might produce significant Ia traffic are largely known, it is currently unclear how NMES parameters such as stimulus intensity, pattern of stimulation and pulse width affect the test results. Therefore, the aim of the present study was to systematically test the effects of different NMES parameters (intensity, pattern of stimulation and pulse width) on the magnitude of the evoked involuntary torque.

## Methods

### Participants

Twenty healthy adults (7 men and 13 women; age: 28.0 ± 5.1 years; body mass: 68.6 ± 15.9 kg; height: 169.8 ± 11.1 cm) volunteered for the study. Exclusion criteria included diagnosed neurological disorders, current acute or chronic injuries, and medications that might affect central monoamine concentrations. Participants were fully informed of any risks or discomforts associated with the procedures before giving their written informed consent to participate. The procedures were approved by the University Human Research Ethics Committee of Edith Cowan University, and performed according to the Declaration of Helsinki, except for registration in a database.

### Procedures

**Fig. 1:**
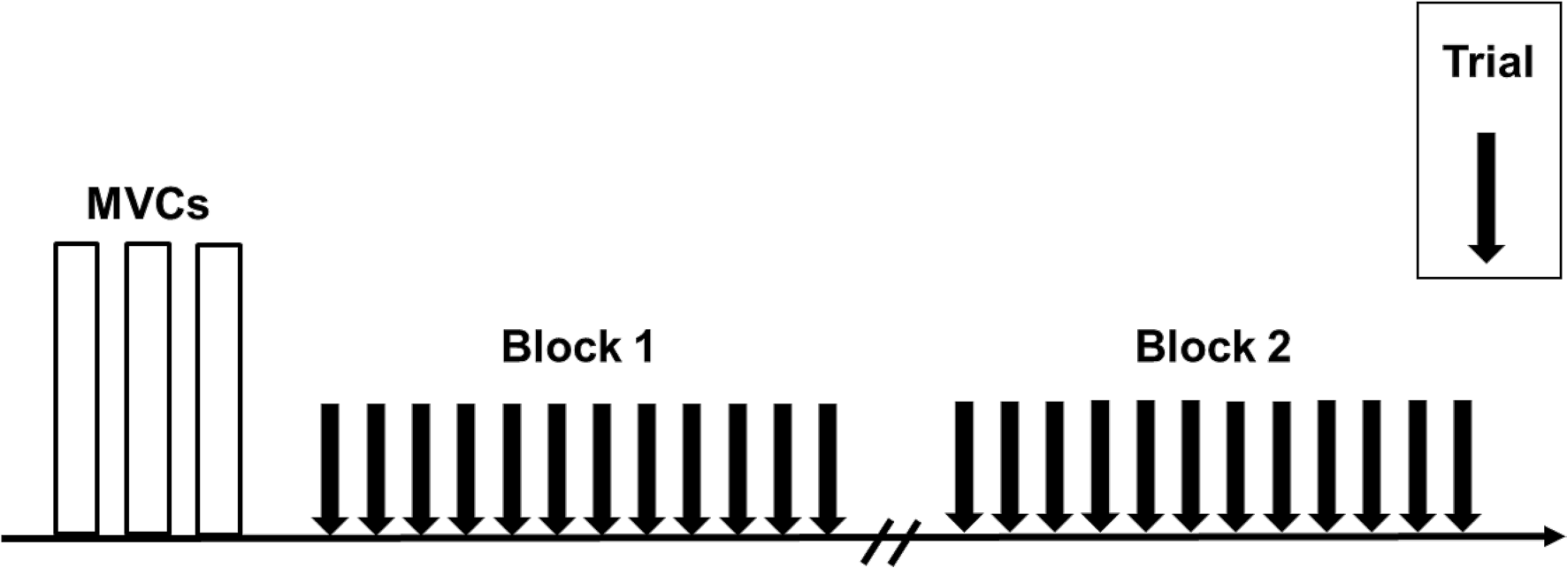
Experimental protocol. After performing maximal voluntary contractions (MVCs) of plantar flexors, a set of up to 12 trials (Block 1) was completed during which plantar flexor neuromuscular electrical stimulation (NMES) was superimposed on Achilles tendon mechanical vibration. A 2nd equivalent block was performed after a 10-min passive rest in those participants who produced a sustained involuntary torque after the cessation of vibration in the trials of Block 1. Between trials, current intensity (to evoke 10, 20 and 30% of MVC torque), pulse width (0.2 ms vs 1 ms) and pattern of stimulation (5 × 2-s bursts at 4-s intervals vs 10 × 1-s bursts at 2-s intervals) were varied randomly

Data collection was performed in a single session and participants were asked to abstain from caffeine-containing foods and beverages within 12 h of the experimental session. Participants were seated in the chair of an isokinetic dynamometer (Biodex System 4, Biodex Medical System, USA) with the hips at 50° of flexion (0° = extended neutral position), right knee fully extended (0°) and right ankle at 10° of dorsiflexion (0° = neutral position). The right foot was tightly fixed to the plantar flexor attachment of the dynamometer and the axis of rotation was aligned with the lateral malleolus. The distance between the chair and the foot attachment was adjusted to find the minimum distance at which participants were able to straighten their right leg, to minimise the elevation of the heel during plantar flexions. Participants were then instructed to perform four isometric plantar flexion contractions at 20, 40, 60 and 80% of perceived maximal effort as warm-up and task familiarization. The participants subsequently performed at least three maximal isometric voluntary plantar flexor contractions (MVC) with a 90-s inter-trial passive rest; additional trials were performed if they reached a torque value > 5% higher than the previous best attempt. Visual plantar flexor torque feedback was continuously provided on a large screen in front of the participant, and standardized verbal encouragement was provided by the same investigator in all trials. The peak isometric plantar flexor torque was taken as the highest value during the MVCs and used to set NMES current intensities. After 3 min of rest, 0.5-s 20-Hz trains of NMES were applied percutaneously to the plantar flexors at increasing intensities to determine the current required to evoke 10, 20 and 30% of MVC torque with both narrow (0.2 ms) and wide (1 ms) pulse widths. Subsequently, NMES was applied in 1- and 2-s bursts with both narrow and wide pulse widths at the pre-determined current intensities during a period of ongoing Achilles tendon vibration in different trials. An initial set of up to 12 trials was conducted, and in each trial a different combination of NMES intensities, pulse widths and patterns was imposed in a randomized order. A warm-up effect during the trial (i.e. increased torque produced in successive stimulations) and self-sustained (ongoing, involuntary) torque following the trial was observable in 11 out of 20 participants. These “responders” then performed a 2nd equivalent block after a 10-min rest. Eight of the 20 participants perceived the intensity to elicit 30% MVC torque as painful (including 3 of 11 “responders”) and therefore trials at this intensity were not conducted for these participants, with only eight trials being performed. Two minutes of rest was provided between trials (Fig. 1).

### Tendon vibration with superimposed NMES

This technique combines tendon vibration and percutaneous NMES to provoke involuntary muscle contractions (Kirk et al. 2019; Magalhães and Kohn 2010; Trajano et al. 2014). The present study was designed to systematically manipulate the NMES parameters used with this technique to determine their influence on involuntary torque variables. In each trial, the Achilles tendon was mechanically vibrated at 115 Hz (1-mm deflections) for 33 s by a hand-held vibrator (Vibrasens, Techno Concepts, France). The vibrator was held with a steady pressure by the same researcher on the posterior aspect of the Achilles tendon at the level of the medial malleolus. After 10 s of vibration, trains of NMES at a 20-Hz frequency were applied to the triceps surae whilst tendon vibration continued. A constant-current electrical stimulator (DS7, Digitimer Ltd, United Kingdom) was used to deliver electrical square-wave stimuli through two self-adhesive electrodes (5 × 9 cm; Dura-Stick Plus, DJO Global, USA), with the cathode placed transversely and distal to the popliteal crease and the anode transversely and over the distal gastrocnemius-Achilles muscle–tendon junction. NMES parameters were varied between trials, with a total of 12 possible combinations. Two pulse widths (0.2 and 1 ms), three current intensities (to evoke 10, 20 and 30% of MVC torque) and two patterns of stimulation (5 × 2-s bursts at 4-s intervals and 10 × 1-s burst at 2-s intervals) were imposed. During the trials, the participants were instructed to hold the shoulder straps of the chair, to look forward at the blank (black) display monitor, silently count backwards from 50 by one, to remain quiet, and to not voluntarily activate their leg muscles.

**Fig. 2:**
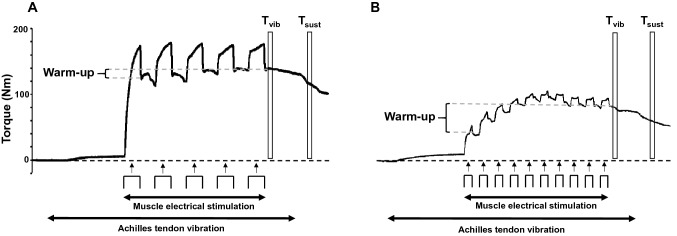
Data from two different trials from the same participant to illustrate the torque response to tendon vibration with superimposed neuromuscular electrical stimulation (NMES). In each trial, the Achilles tendon was mechanically vibrated at 115 Hz for 33 s. After 10 s of vibration, NMES (20 Hz) was applied to the triceps surae whilst tendon vibration continued. Torque produced during a test using 5 × 2-s bursts at 4-s intervals (2-s on, 2-s off) with a wide (1-ms) pulse width and at an intensity that evoked a torque of 20% of maximal voluntary contraction (MVC) torque is shown in A, and torque produced using 10 × 1-s bursts at 2-s intervals (1-s on, 1-s off) with a narrow (0.2-ms) pulse-width and at an intensity that evoked a torque of 10% MVC is shown in B. Current intensity, pulse width and pattern of stimulation were altered in each trial. *T*_vib_ torque measured during vibration after the last burst of NMES, *T*_sust_ torque measured 3 s after vibration cessation (self-sustained torque), *Warm-up* difference between T_vib_ and torque after the first burst of NMES

Three torque-related quantities were calculated (Fig. 2) and presented as a percentage of MVC.

*Reflexive torque during vibration *(*T*_vib_): mean torque in a 500-ms window starting 500 ms after the cessation of NMES, but during tendon vibration.

*Self-sustained torque *(*T*_sust_): mean torque in a 500-ms window starting 3 s after the cessation of vibration.

*Warm-up:* the difference between *T*_vib_ and the mean torque in a 500-ms window starting 500 ms after the first burst of NMES.

### Statistical analysis

Data are presented as mean ± standard deviation (mean ± SD). Statistical analyses were performed using IBM SPSS Statistics (v25, SPSS Inc., USA). Normality of the data was examined using Shapiro–Wilk test. Warm-up effect, *T*_vib_ and *T*_sust_ were subjected to separate 2 (Intensity: 10 vs 20% MVC) × 2 (Pulse width: wide vs narrow) × 2 (Pattern: 5 × 2-s vs 10 × 1-s bursts) × 2 (Block: block 1 vs block 2) repeated measures ANOVA. Given that not all participants performed 30% MVC trials, statistical analysis was initially conducted without the 30% MVC condition (*n* = 11). Additional ANOVAs with the inclusion of this intensity were also conducted (*n* = 8). For all ANOVAs, sphericity was assessed using Mauchly’s test of sphericity; Greenhouse–Geisser correction was used when sphericity was violated. The Least Significant Difference method was used for post-hoc comparisons when up to three means were compared, whilst a false discovery rate analysis was used when more than three means were compared to minimise the risk of type I error resulting from multiple pairwise comparisons. Effect sizes are reported as partial eta squared (ƞ_p_^2^). Statistical significance was set at an alpha level of 0.05.

Follow-up pairwise analysis of trials for reliability was performed for the combination of electrical parameters which yielded the greatest magnitude of response. The level of reliability of the involuntary torque variables was determined when assessed in different sets (i.e. Set 1 vs Set 2). The indices of reliability that were calculated were two-way mixed effects intraclass correlation coefficients (ICC _3,1_), typical error (TE; i.e. standard error of measurement) through a freely available spreadsheet (Hopkins 2017), minimum detectable change (MDC) through the formula MDC = TE × 1.96 × √2, and intra-individual coefficient of variation (CV) as the average of the CVs for each individual.

## Results

**Fig. 3:**
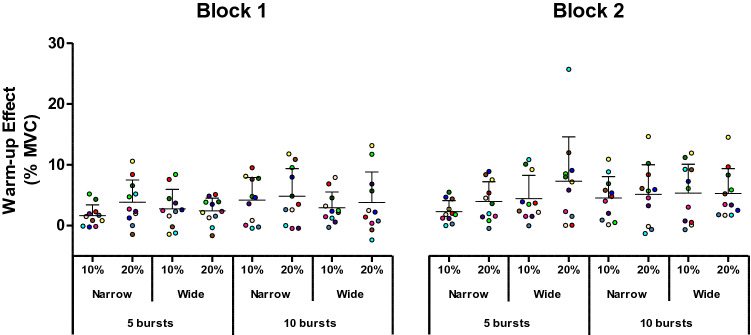
Magnitude of warm-up in the different conditions. Trains of neuromuscular electrical stimulation (NMES) were applied percutaneously to the plantar flexors with a current intensity to evoke 10 or 20% of maximal voluntary contraction (MVC) torque, with narrow (0.2 ms) or wide (1 ms) pulse widths, in 5 × 2-s bursts at 4-s intervals, or 10 × 1-s bursts at 2-s intervals. Symbols represent data from individuals (*n* = 11), normalized to MVC. Means are shown in horizontal lines with standard deviations indicated. A significant main effect of “intensity” in the ANOVA revealed that a stimulation intensity that evoked 20% of MVC torque produced a larger warm-up effect than an intensity that evoked 10% MVC. A significant interaction effect of NMES and pulse width revealed a lower warm-up effect with 5 bursts of narrow pulse-width compared to 10 bursts of narrow pulse-width stimulation

**Fig. 4:**
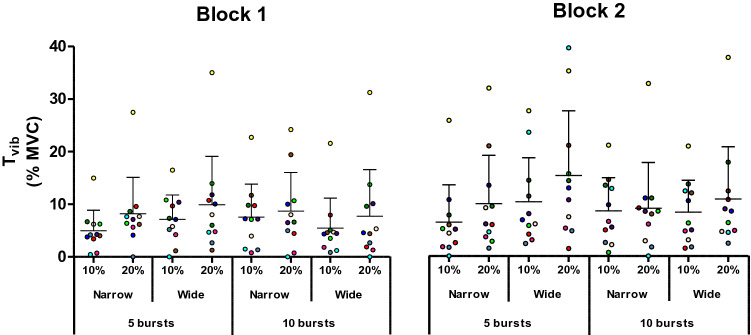
Magnitude of the reflexive torque during vibration (*T*_vib_) in the different conditions. Trains of NMES were applied percutaneously to the plantar flexors with a current intensity to evoke 10 or 20% of maximal voluntary contraction (MVC) torque, with narrow (0.2 ms) or wide (1 ms) pulse widths, in 5 × 2-s bursts at 4-s intervals, or 10 × 1-s bursts at 2-s intervals. Symbols represent data from individuals (*n* = 11), normalized to MVC. Means are shown in horizontal lines with standard deviations indicated. A significant main effect of “intensity” in the ANOVA revealed that a stimulation intensity that evoked 20% of MVC torque produced a larger *T*_vib_ than an intensity that evoked 10% MVC. A significant interaction effect of NMES and pulse width revealed a higher *T*_vib_ being evoked by 5 bursts of wide pulse-width stimulation than by 5 bursts of narrow pulse-width or by 10 bursts of wide pulse-width stimulation

**Fig. 5:**
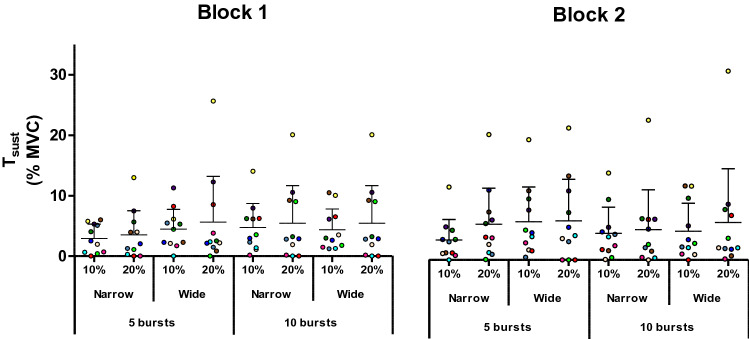
Magnitude of the sustained torque (*T*_sust_) in the different conditions. Trains of NMES were applied percutaneously to the plantar flexors with a current intensity to evoke 10 or 20% of maximal voluntary contraction (MVC) torque, with narrow (0.2 ms) or wide (1 ms) pulse widths, in 5 × 2-s bursts at 4-s intervals, or 10 × 1-s bursts at 2-s intervals. Symbols represent data from individuals (*n* = 11), normalized to MVC. Means are shown in horizontal lines with standard deviations indicated. A significant main effect of “pulse width” in the ANOVA revealed that wide-pulse NMES evoked a greater *T*_sust_ than narrow-pulse NMES

Both a warm-up effect during the trial and self-sustained torque following vibration cessation were observed in 11 out of 20 participants (6 of 7 men and 5 of 13 women). These 11 participants were designated as “responders” and formed the study cohort. Trials with NMES intensity of 30% MVC were not performed by all participants, as 8 out of 20 (3 out of 11 responders) perceived this intensity as painful. Hence, analyses were initially conducted without the 30% MVC condition (*n* = 11), with the results being presented below. Individual data under different experimental conditions can be seen in Fig. 3 (warm-up effect), Fig. 4 (*T*_vib_) and Fig. 5 (*T*_sust_).

Significant effects of intensity on both warm-up [F(1,10) = 8.283, *p* = 0.016, *ƞ*_p_^2^ = 0.453] and *T*_vib_[F(1,10) = 9.006, *p* = 0.013, *ƞ*_p_^2^ = 0.474] were observed, with both variables being greater in the 20% MVC than 10% MVC condition (*T*_vib_: 9.7 ± 9.0 vs 7.1 ± 6.1% MVC, *p* = 0.015; warm-up effect: 4.3 ± 4.5 vs 3.7 ± 3.5% MVC, *p* = 0.016). No significant effect of intensity was observed for *T*_sust_ (5.3 ± 6.2 vs 4.4 ± 3.9% MVC, *p* = 0.364).

Pulse width significantly affected *T*_sust_ [F(1,10) = 6.093, *p* = 0.033, *ƞ*_p_^2^ = 0.379] but not warm-up (1-ms vs 0.2-ms width: 4.2 ± 4.5 vs 3.7 ± 3.6% MVC, *p* = 0.349) or *T*_vib_ (9.2 ± 8.6 vs 7.8 ± 7.0% MVC, *p* = 0.107). *T*_sust_ was higher in the 1-ms than 0.2-ms pulse-width condition (5.3 ± 5.9 vs 4.1 ± 4.3% MVC, *p* = 0.033).

No effect of stimulation pattern (5 × 2-s vs 10 × 1-s bursts) was observed on any of the involuntary torque variables (*T*_vib_: *p* = 0.267, *T*_sust_: p = 0.925, warm-up effect: *p* = 0.239). However, a significant interaction between pattern of stimulation and pulse width was observed for the warm-up effect [F(1,10) = 5.143, *p* = 0.047, *ƞ*_p_^2^ = 0.340] and *T*_vib_ [F(1,10) = 11.799, *p* = 0.006, ƞ_p_^2^ = 0.541]. A significantly higher *T*_vib_ was evoked by 5 bursts of wide pulse-width stimulation (10.6 ± 9.1% MVC) than by 5 bursts of narrow pulse-width (7.4 ± 7.0% MVC, *p* = 0.003) or by 10 bursts of wide pulse-width (8.0 ± 7.8% MVC, *p* = 0.012). Moreover, a significantly smaller warm-up effect was observed with 5 bursts of narrow pulse-width compared to 10 bursts of narrow pulse-width stimulation (2.9 ± 2.8% vs 4.7 ± 4.1% MVC, *p* = 0.007). No other significant interactions were observed.

**Fig. 6:**
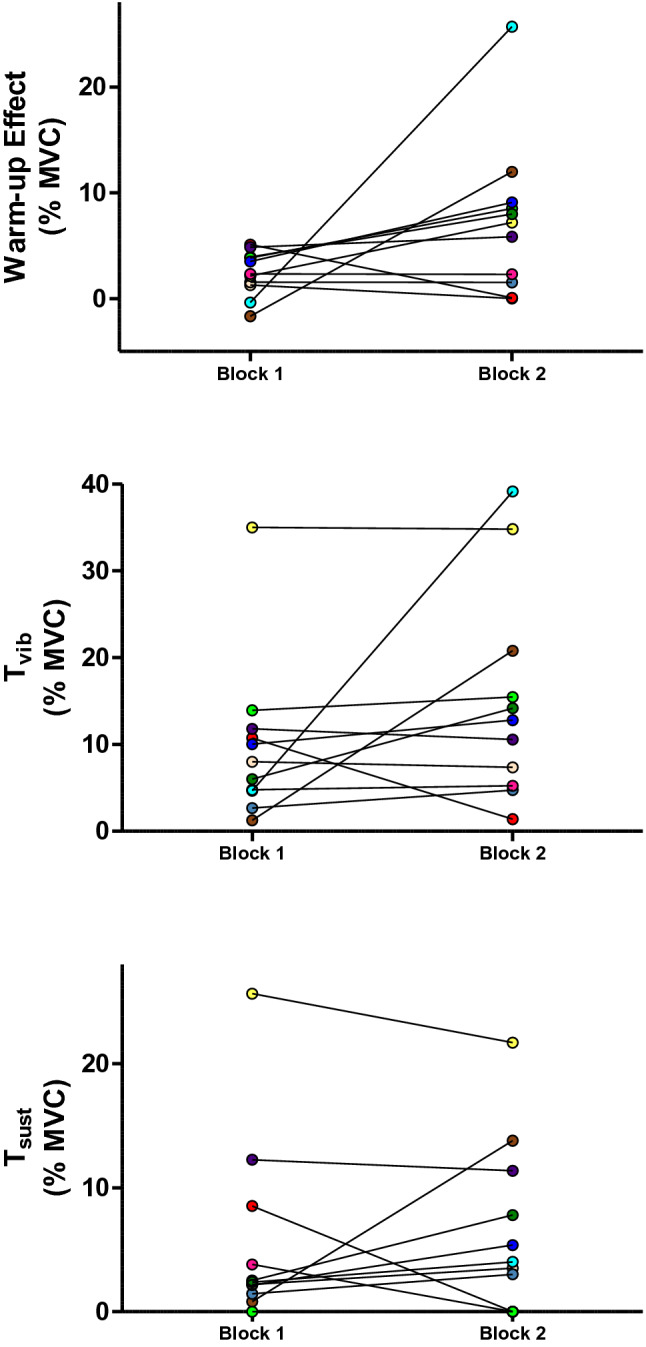
Individual data of warm-up effect, reflexive torque during vibration (*T*_vib_) and sustained torque (*T*_sust_) for the two blocks. Symbols represent data from individuals (*n* = 11), normalized to the maximal voluntary contraction torque (MVC), and are shown for the condition which evoked greatest responses. In this condition, trains of neuromuscular electrical stimulation were applied percutaneously to the plantar flexors with a current intensity to elicit 20% of MVC torque, with wide (1 ms) pulse-width, in 5 × 2-s bursts at 4-s intervals. Analyses of variance did not reveal a significant difference between blocks for any of the variables, when all the independent factors were taken into consideration. Nevertheless, a high degree of variability was observed in some of the participants

Overall, the greatest responses were observed in the condition of 5 × 2-s bursts at 4-s intervals with a wide (1 ms) pulse-width and at an intensity that evoked a torque of 20% MVC. However, in spite of an absence of a significant difference between sets for any variable (*T*_vib_: *p* = 0.094, *T*_sust_: *p* = 0.234, warm-up effect: *p* = 0.109), the level of reliability for this combination of electrical parameters between Set 1 and Set 2 was low (Table 1), with a high degree of variability being observed in some of the participants (Fig. 6).

**Table 1:** Reliability statistics for differences between Set 1 and Set 2 in the condition of 5 × 2-s bursts at 4-s intervals, with a wide (1 ms) pulse-width, and at an intensity that evoked a torque of 20% MVC

	TE (95% CI) (%MVC)	CV%	MDC (%MVC)	MDC (%)	ICC (95%)
Warm-up	6 (4–11)	78.37	27	553.61	− 0.2 ( – 0.569–0.365)
*T*_vib_	4 (3–7)	58.16	15	273.36	0.178 ( – 0.432–0.682)
*T*_sust_	4 (3–7)	53.39	17	115.73	0.716 (0.232–0.915)

*TE* typical error, *CV* coefficient of variation, *MDC* minimum detectable change, *ICC* intraclass correlation coefficient, *T*_*vib*_ and torque after the first burst of NMES, *T*_*sust*_ torque measured 3 s after vibration cessation (self-sustained torque)

Analyses of variance with the inclusion of 30% MVC trials (*n* = 8) only reached significance for *T*_vib_, with a significant main effect of intensity [F(1.167,8.171) = 6.881, *p* = 0.027, *ƞ*_p_^2^ = 0.496]. *T*_vib_ was significantly lower in the 10% trials (6.3 ± 4.4%) than 20% (7.9 ± 6.2%, *p* = 0.011) and 30% MVC trials (9.7 ± 7.3%, *p* = 0.038), with no significant difference between the 20 and 30% MVC trials (*p* = 0.507).

## Discussion

The aim of the present study was to identify the optimal NMES parameters for use in a method that combines NMES and tendon vibration to evoke involuntary torque. The NMES parameters of intensity, pulse width and pattern of stimulation were systematically tested to determine their effect on the magnitude of increased torque in response to repeated bursts of NMES, the involuntary torque after NMES while the tendon was vibrated, and the sustained torque produced after cessation of tendon vibration. The greatest responses were found with the use of 2-s bursts of NMES every 4 s (i.e. 2-s rest between bursts) with a wide (1-ms) pulse-width and at an intensity that evoked a torque of 20% MVC.

In the present study, wide-pulse (1 ms) NMES evoked a greater *T*_sust_ than narrow-pulse (0.2 ms) NMES. These results are consistent with previous findings of greater levels of “extra torque” (Collins et al. 2002; Lagerquist and Collins 2010) and EMG activity (Arpin et al. 2019) during wide- than narrow-pulse NMES. As the membranes of sensory axons have a longer strength-duration time constant and lower rheobase than motor axons (Veale et al. 1973), a higher Ia afferent bombardment of the MN is likely in the wide-pulse conditions, which probably led to a greater MN activity.

Stimulation that evoked 20% of MVC torque produced a larger warm-up effect and *T*_vib_ than stimulation that evoked 10% MVC. Increasing the current intensity applied over the muscle belly produces a stronger depolarising stimulus, which should activate more sensory axons, enhancing the reflex synaptic input to the MN (Zehr 2002). However, increasing current intensity to evoke 30% MVC did not further augment the observed responses. Speculatively, this might have resulted from greater antidromic collision (Gottlieb and Agarwal 1976; Gregory and Bickel 2005), or from a pain-induced inhibition of MN activity and/or PIC strength (Lund et al. 1991; Farina et al. 2004; Sandrini et al. 2005; Revill and Fuglevand 2017). Taking into consideration the low number of responders who tolerated the 30% MVC trial in the current study (*n* = 8), further research should confirm that there is no further advantage in increasing intensity to a target level of 30% MVC in those who tolerate it well.

Interrogation of the interaction effect between pattern of stimulation and pulse width revealed that 5 × 2-s bursts were superior to 10 × 1-s bursts. This is consistent with the findings of Dean et al. (2007), who reported a greater “extra torque” as burst duration increased from 0.25 to 2 s. One possibility is that longer bursts may have allowed a cumulative excitatory effect of Ia afferent bombardment onto the MN, which then recruited more MNs and allowed PICs to grow within each burst. An enhancement of PIC strength within each burst could reflect the slow activation kinetics (Lee and Heckman 1998) and warm-up (Svirskis and Hounsgaard 1997) exhibited by PICs carried by calcium ions. Moreover, given that a torque plateau was not reached in the 2-s condition (Fig. 2), future studies could potentially investigate whether a longer duration of electrical stimulation with fewer bursts would yield similar, or higher, levels of involuntary torque.

It is important to note that the combination of electrical parameters that we recommend evoked responses with greater magnitude but had low levels of reliability between blocks (i.e. between two non-consecutive trials), with the warm-up effect showing the lowest reliability. By contrast, Trajano et al. (2014) showed high levels of reliability of the involuntary torque variables when two trials were performed in succession. The discrepancy between the current results and the findings by Trajano et al. (2014) may be attributed to the performance of 8 or 12 trials, each with different stimulation parameters, between the trials of interest. Numerous factors could contribute to a change in the involuntary torque variables throughout the protocol (as observed in some of the participants, Fig. 6), such as warm-up of PIC amplitude (Svirskis and Hounsgaard 1997), muscle peripheral fatigue (Mettler et al. 2018), decreased efficacy of the Ia afferent-α-motor neurone pathway (Pope and DeFreitas 2015) and a change in the mental state of the individual throughout the protocol [i.e. more stressed or more relaxed, (Collins et al. 2002)]. While some of these confounding factors were potentially minimised by the 90-s inter-trial and 10-min inter-block passive rests, their influence cannot be ruled out.

A sustained involuntary torque after the cessation of vibration was not observed in all participants, and some responders showed low levels of involuntary torque. The observation of responders and non-responders has been previously reported in other human studies in which a combination of tendon vibration and NMES (Kirk et al. 2019; Magalhães and Kohn 2010) or NMES only (e.g. Wegrzyk et al. 2015) was imposed. The physiological explanation for this high inter-individual variability is unknown. However several possible mechanisms can be hypothesized, including differences in vibration propagation (Guang et al. 2018), through differences in excitability of the Ia reflex circuitry (Crone and Nielsen 1989; Maffiuletti et al. 2008; Macefield and Knellwolf 2018), to differences in spinal levels of monoamines (Collins 2007; Dean et al. 2007), and the level of relaxation or stress of the participants. However, it should be noted that more non-responders were found in the female cohort, which contrasts the similar proportions of male and female responders observed by Wegrzyk et al. (2015). The potentially higher percentage of female non-responders should be verified in future studies and the possible causes investigated.

Mechanisms apart from activation of PICs in the MNs may have contributed to the observed involuntary torque enhancement during the test, including pre-synaptic post-tetanic potentiation of the Ia input to the MNs (Hirst et al. 1981) and modulation of intrinsic muscle properties (Frigon et al. 2011; Blazevich and Babault 2019). However, these mechanisms are unlikely to explain the self-sustained torque in the current study and ongoing soleus EMG activity observed in previous studies (Trajano et al. 2014; Kirk et al. 2019), after the cessation of tendon vibration. One could argue that sustained MN firing could have been caused by the activity in primary muscle spindle afferents as a consequence of thixotropic properties of intrafusal muscle fibres (Kuffler et al. 1951). However, in this technique, a spindle post-contraction sensory discharge is unlikely given that there is no significant background fusimotor drive to spindle endings in the absence of voluntary drive (Gandevia et al. 1986). By contrast, activation of PIC channels to cause bistable behavior in some spinal MNs (Lee and Heckman 1998) is a plausible mechanism to explain the observation of sustained involuntary muscle contractions in 11 out of 20 participants after the cessation of tendon vibration. Similarly, the observation of a warm-up effect caused by repetitive activation (i.e. progressive increase in torque within the trial) is also consistent with the activation of PICs in the present protocol (Svirskis and Hounsgaard 1997). Nevertheless, the contribution of PICs is currently unproven. With the advent of new high-density surface EMG techniques, future research could examine the firing of individual MUs during ongoing production of involuntary torque with this technique and potentially examine whether self-sustained firing of MNs, denoting bistable behavior after the cessation of vibration, can be observed (Lee and Heckman, 1998). Moreover, it would be interesting to systematically compare potential changes in the involuntary torque production using this technique with potential changes in ∆F values of the paired motor unit technique (Gorassini et al. 2002) during voluntary contractions, as the latter technique is presently the standard method to indirectly estimate PIC strength in humans. To date, acute static stretching is one intervention that has been observed to reduce both involuntary plantar flexion torque production (Trajano et al. 2014) and soleus ∆F values (Trajano et al. 2020), indicating that both techniques may provide similar information. If tendon vibration and NMES can be established as a measure of PICs, it should be noted that not everyone responds to the stimulus and some participants have a low magnitude of involuntary torque, which might impact the widespread use of this methodological approach.

A limitation of the current study is that the Achilles tendon was mechanically vibrated at 115 Hz with 1-mm deflections by an experienced researcher, but the pressure on the skin was not quantified. It is possible that small pressure variations might have partly contributed to the observed variability between blocks. Finally, future research could quantify whether higher-frequency trains of NMES would have resulted in involuntary torque with higher amplitude (Dean et al. 2007), given that it might further enhance the effective current delivered to MNs along the sensory axons. However, higher stimulation frequencies may both increase the risk of peripheral fatigue during a long experimental protocol (Grosprêtre et al. 2017) and variably influence neurotransmitter release from Ia afferent terminals (Hirst et al. 1981; Crone and Nielsen 1989).

## Conclusion

A combination of Achilles tendon vibration with superimposed NMES on triceps surae induces involuntary sustained torque in some individuals. Tailoring NMES parameters modulates the magnitude of the involuntary torque, and we observed that a current intensity to evoke 20% of MVC torque with wide (1 ms) pulse-width in 5 × 2-s bursts and at 4-s intervals produces the greatest responses and therefore is recommended for use with this technique. These findings may also inform the parameters of percutaneous electrical stimulation used in other clinical and experimental settings.

## Data Availability

Non-identifiable datasets generated and/or analyzed during the current study are available from the corresponding author on reasonable request.

## Abbreviations

CV: Coefficient of variation
EMG: Electromyographic
ICC: Intraclass correlation coefficient
MU: Motor unit
NMES: Neuromuscular electrical stimulation
MDC: Minimum detectable change
MNs: Spinal motor neurones
MVC: Maximal isometric voluntary plantar flexor contraction
PICs: Persistent inward currents
TE: Typical error
*T*_sust_: Self-sustained torque
*T*_vib_: Reflexive torque during vibration
WU: Warm-up
*ƞ*_p_^2^: Partial eta-squared

## Code availability

Macros for semi-automated analysis of torque data in LabChart utilized in this study are available from the corresponding author on reasonable request.
